# Abdominal wall abscess after cholecystectomy

**DOI:** 10.1186/s13104-015-1303-9

**Published:** 2015-08-05

**Authors:** Fabian Grass, Ian Fournier, Vincent Bettschart

**Affiliations:** Department of General Surgery, Hôpital du Valais, Av. Grand-Champsec 80, 1951 Sion, Switzerland

**Keywords:** Abscess, Gallstone, Cholecystectomy

## Abstract

**Background:**

Laparoscopic cholecystectomy is one of the most frequently performed surgical interventions nowadays in developed countries. While lost gallstones during the procedure represent a commonly encountered issue, there is an ongoing debate whether split gallstones imperatively need to be extracted during the same procedure. The reported case of a wall abscess several years after follow-up lights up this debate.

**Case presentation:**

A 75-year-old male Caucasian with a history of rheumatoid arthritis and congestive heart failure presented with a recurrent subcutaneous abdominal wall abscess with occasional, spontaneous drainage of pus. He underwent laparoscopic cholecystectomy for acute calculous cholecystitis 3 years ago with uneventful and prompt recovery. A computed tomography scan showed a cavity in the periumbilical abdominal wall with peripheral contrast-enhancing, next to a calcified foreign body between the rectus muscle sheets. Wound exploration under general anaesthesia was performed with drainage of the cavity, extraction of the foreign body and closure of the anterior rectus sheet over a drainage catheter. The foreign body turned out to be a gallstone lost in the periumbilical port site during the procedure. Antibiotic treatment with co-amoxiclav was continued for 14 days. The patient was discharged 9 days postoperatively with a clean wound.

**Conclusion:**

This case and short review of the literature is a reminder of the importance of careful extraction of split gallstones during cholecystectomy in order to avoid early or late complications. This is especially important in the light of one of the most commonly performed surgical procedures in developed countries with generally low morbidity.

## Background

Split gallstones during laparoscopic cholecystectomy are a well known issue when the gallbladder containing the calculus is accidentally perforated during the procedure. An Australian group reviewed eight studies with over 18,000 performed laparoscopic cholecystectomies and found an incidence of perforation of the gallbladder of 18.3% (8–39.9%) and an incidence of spilt gallstones of 7.3% (0.1–20%), while one third (5–48%) of the split gallstones were left unretrieved [1]. Whether unretrieved gallstones cause damage has been studied and remains somehow controversial, since some authors found an increased risk of intraabdominal adhesion and abscess formation while others describe no consequences unless the stones are crushed or associated with acutely inflamed gallbladder [2, 3]. The aim of the present report is to remind of the potential late consequences of unretrieved gallstones within the peritoneal cavity or abdominal wall.

## Case presentation

**Fig. 1:**
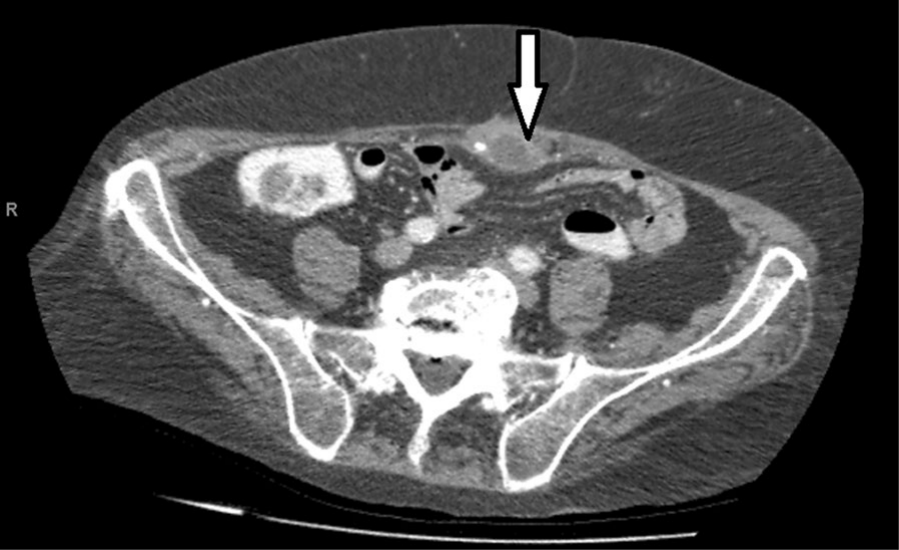
Foreign body on computed tomography scan. The *arrow* highlights the contrast-enhancing abscess cavity next to a calcified foreign body.

A 75-year-old male Caucasian with a history of rheumatoid arthritis and congestive heart failure presented with a recurrent subcutaneous abdominal wall abscess with occasional, spontaneous drainage of pus. He underwent laparoscopic cholecystectomy for acute calculous cholecystitis 3 years ago. Physical examination revealed periumbilical redness and tenderness with a draining percutaneous tract. Laboratory testing revealed a white cell count of 13,900 per cubic millimetre. A computed tomography (CT) scan showed a cavity in the periumbilical abdominal wall with peripheral contrast-enhancing, next to a calcified foreign body between the rectus muscle sheaths (Fig. 1, arrow). Wound exploration under general anaesthesia was performed with drainage of the cavity, extraction of the foreign body and closure of the anterior rectus sheet over a drainage catheter. On pathological examination, the foreign body turned out to be a gallstone. It was lost in the periumbilical port site during the procedure in which gallbladder perforation occurred. “*E. coli*” bacteria were found on microbiological array. Antibiotic treatment with co-amoxiclav was continued for 14 days. The patient was discharged 9 days postoperatively with a clean wound, and continued to visit the outpatient clinic for wound follow-up for 8 weeks. Follow-up was uneventful.

## Discussion

Since over 90% of split gallstones never become symptomatic, they often present as incidental findings on CT-scans. Particular locations such as Morison’s pouch or even intrathoracic stones have been described [4, 5]. “Loose bodies” are prone to change place on diagnostic imaging and represent a diagnostic challenge [4]. It has been reported that 8.5% of lost gallstones will lead to a complication, and risk factors such as acute cholecystitis with infected bile, pigment stones, prone to higher bacterial contamination, multiple stones (>15), the stone size (>1.5 cm) and age have been described [6]. Careful removal of as many stones as possible, intense irrigation and suction (10 mm device) and avoidance of spread into difficult accessible sites, as well as the use of intraabdominal bags and laparoscopic graspers are recommended [6]. Stones should routinely be examined for infection and treated accordingly.

Informed patient consent with mentioning of this potential late complication might help to avoid misunderstandings later on. Of note, interventional abscess puncture without stone removal implies a high risk of recurrence [6].

## Conclusion

The recently encountered case of an abdominal wall abscess due to an infected gallstone, split during a surgical intervention several years ago, is a reminder of this displeasing late complication. It reminds the surgical community of the importance of prompt and careful removal of these stones, especially when confronted with acute cholecystitis. Treating physicians confronted with unusual abscess locations should bear in mind this rare complication when confronted with an unusual abscess formation in patients who have previously undergone laparoscopic gall bladder removal.

## Consent

Written informed consent was obtained from the patient for publication of this Case Report and any accompanying images. A copy of the written consent is available for review by the Editor-in-Chief of this journal.
